# Biocompatibility of A Nano-curcumin Pulpal Paste in Rats: Biocompatibility of a Nano-curcumin Paste

**DOI:** 10.31661/gmj.v13iSP1.3579

**Published:** 2024-12-08

**Authors:** Rasoul Sahebalam, Alireza Sarraf Shirazi, Narges Ghazi, Mahshid Gifani, Berahman Sabzevari

**Affiliations:** ^1^ Pediatric Dentistry Department, Dental School, Mashhad University of Medical Sciences, Mashhad, Iran; ^2^ Oral and Maxillofacial Pathology Department, Dental School, Mashhad University of Medical Sciences, Mashhad, Iran; ^3^ Orthodontist, Private Practice, Mashhad, Iran

**Keywords:** Materials Testing, Curcumin, Metapex, Rats, Tooth, Deciduous

## Abstract

**Background:**

This study aimed to assess the biocompatibility of different concentrations of a nano-curcumin pulpal paste in rats.

**Materials and Methods:**

Polyethylene tubes containing zinc oxide eugenol (ZOE), Metapex, and 2, 4, 6, and 8 ppm nano-curcumin pulpal paste, and an empty tube as the negative control were implanted in the back of 30 Wistar rats (7 tubes per each rat). The rats were sacrificed after 15, 30, and 60 days (10 rats at each time point). The tissue around the tubes underwent histopathological analysis. After hematoxylin and eosin staining, the specimens were evaluated for presence/absence of necrosis and calcification, number of inflammatory cells, and thickness of soft tissue capsule. Data were analyzed by the Chi-square, Mann-Whitney, and Kruskal-Wallis tests (α=0.05).

**Results:**

Necrosis was not seen in any group at any time point. No significant difference existed among the experimental groups regarding calcification at different time points (P0.05). The fibrotic capsule was thin in all experimental groups at all time points. Rate of inflammation decreased in all experimental groups from day 15 to day 60. Among different concentrations, only 2 ppm concentration of nano-curcumin paste had no significant difference with Metapex and ZOE regarding inflammation at different time points.

**Conclusion:**

All tested concentrations of nano-curcumin pulpal paste were biocompatible, compared with the positive controls (ZOE and Metapex); but 2 ppm concentration was the most biocompatible. Within the limitations of this in vitro study, 2 ppm concentration of nano-curcumin may be suggested for further experiments.

## Introduction

Pulp therapy is commonly performed for primary teeth with extensive caries, traumatic exposure, structural defects, or pulp involvement and includes pulpectomy, pulpotomy, direct pulp capping, and indirect pulp capping [1]. Success of pulpectomy depends on precise conduction of the procedural steps (i.e., access cavity preparation, debridement, elimination of inflamed pulp tissue, root canal irrigation and filling, and proper coronal restoration), and use of suitable biocompatible materials [2]. Application of a root filling material after elimination of the infected pulp tissue is an important step in success of pulp therapy. An ideal root canal filling material should be antiseptic, radiopaque, non-irritant for the underlying tooth germ, non-toxic, and biocompatible. It should also have easy handling, optimal flow in the complex root canal system, easy retrieval, and a resorption speed similar to the speed of physiological resorption of primary root. Also, it should be easily resorbed if passed through the apex, have insignificant shrinkage, and provide optimal seal [3][4]. None of the available root filling materials have all the aforementioned criteria. Thus, research is still ongoing in this regard [3]. Zinc oxide eugenol (ZOE) is the most commonly used root filling material in primary teeth [5]. However, it has some drawbacks. It has a resorption rate slower than the rate of physiological resorption of primary root. Also, if extruded through the primary root apex, it converts to a hard cement, which is resistant to resorption. ZOE residues are detected in 94% of pulpectomized teeth, which may remain in the alveolar bone for months to years, and change the eruption path of permanent successors [3]. Also, ZOE is irritant for the tissue, and can cause foreign body reaction at the peri-apex. Although eugenol in its composition has analgesic properties, it is stimulant as well [6]. Moreover, ZOE has limited antimicrobial activity [5].

Calcium hydroxide (CH) pastes with iodoform (such as Metapex) are also used for root canal filling of primary teeth. The antimicrobial activity of CH is due to its calcium ions. Aqueous, viscous, or oily carriers are used in the formulation of root canal filling pastes, which affect the release of ions. For instance, aqueous carriers are highly soluble, resulting in fast resorption of paste before the physiological resorption of primary tooth root. Viscous carriers have a lower solubility, and oily carriers have the lowest solubility, enabling better release of CH. Pastes containing CH and iodoform such as Metapex contain 30.3% CH, 40.4% iodoform, 22.4% silicone oil, and 6.9% other ingredients. Metapex is radiopaque and premixed, and is easy to apply. If extruded through the apex, it is resorbed within 1 to 2 weeks. A previous study showed that pulpectomy with ZOE, Metapex, and Vitapex yielded similar results and led to bone regeneration as confirmed clinically and histologically [3]. However, iodoform-based pastes can cause yellowish-brown discoloration of teeth and compromise esthetics [5].

Currently, use of medicinal herbs is on the rise since it is believed that they have optimal therapeutic properties with lower side effects than synthetic medications. Curcumin is the effective substance of turmeric and has unique health benefits. Its rhizome has antioxidant, antibacterial, anti-inflammatory, and anti-cancer properties [7][8][9][10][11][12][13]. Curcumin is a hydrophobic polyphenol derived from the rhizome of turmeric [8]. Curcumin is insoluble in water and ether, and soluble in ethanol, dimethyl sulfoxide, and acetone [9]. However, despite its unique properties, some shortcomings exist with respect to the use of curcumin in the formulation of medications, such as its low water solubility, photodegradation, chemical instability at the physiological pH, fast metabolism, short shelf-life, and low bioavailability [14]. Use of nano-carriers is one suggested strategy to improve the poor biopharmaceutical properties of curcumin. Nano-technology has been employed to improve the solubility of lipophilic substances such as curcumin, maximize their bioavailability and enhance their distribution. Evidence shows that nano-carriers are effective to improve the bioavailability of curcumin [15][16][17][18].

The chemical composition of nano-curcumin is similar to that of curcumin but nano-curcumin has significantly higher water solubility and antimicrobial activity due to the reduction in particle size [16][19]. Also, the anti-inflammatory effects of curcumin are enhanced by the improved absorption of nano-curcumin. Thus, topical application of nano-curcumin may have effects compatible to the effects of systemic use of curcumin [15]. Nanomicelles are a type of nano-formulation to improve the biological properties of curcumin. This technology is effective for encapsulation of medications with low solubility. Nanomicelles range in size from 20 to 100 nm. Easy production, low cost, easy drug delivery through the biological barriers, increased solubility in aqueous environments and water, and protection of medication against degradation are among the advantages of nanomicelles [14]. Biocompatibility refers to the ability of a material to induce the desired biological response with no or minimal inflammatory reaction in the viable tissues. To assess biocompatibility, the material is often implanted subcutaneously in animals [20]. Since root filling materials are in direct contact with the surrounding viable tissues such as the pulp and periapical tissues and periodontal ligament, they must be biocompatible.

Considering the optimal properties of nano-curcumin and its potential for use in pulp therapy of primary teeth, this study aimed to assess the biocompatibility of different concentrations of a nano-curcumin pulpal paste in rats.

## Materials and Methods

**Figure-1:**
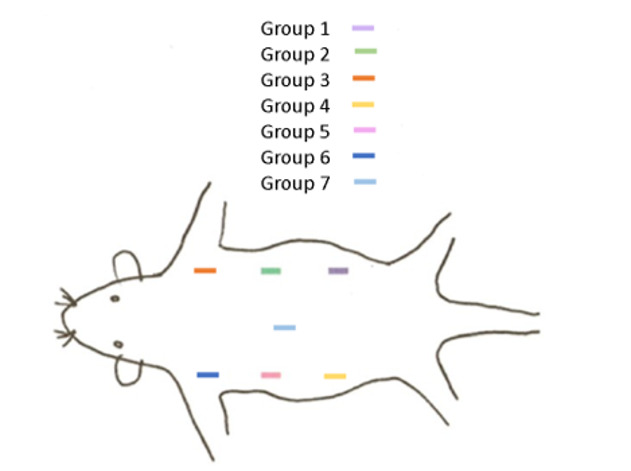
Figure 1. Order of implanting 7 polyethylene tubes in the back of each rat

**Figure-2:**
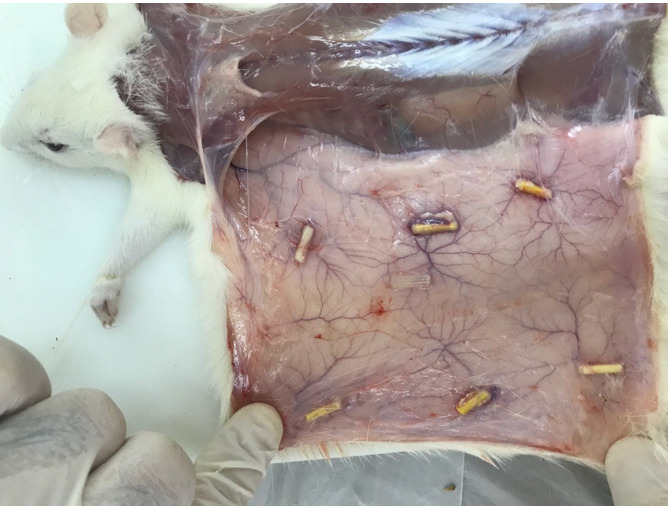
Figure 2.Resection of specimens after sacrificing the rats

This study was conducted on 30 adult male Wistar rats weighing 200 to 220 g. The study protocol was approved by the ethics committee of Mashhad University of Medical Sciences (IR.mums.sd.REC.1394.315), and implemented in accordance with the guidelines for the care and use of laboratory animals.

### Sample Size

The sample size was calculated to be 9 rats in each group according to a study by Scarparo et al, [21] assuming alpha=0.05, and study power of 80% using the formula for the comparison of means of two independent variables. To increase the reliability of the results, 10 rats were evaluated in each group.

### Preparation of Nano-curcumin

Curcumin was obtained in nanomicelle form from the Nanotechnology Research Center of Mashhad University of Medical Sciences, and weighed with a digital scale (KEM; Kia Electronic Pars, Iran) with 0.001 g accuracy.

Sterile polyethylene tubes (Supa, Iran) with 10 mm length and 1 mm internal diameter were used for this study [22][23][24][25]. Seven tubes were considered for each rat containing Metapex (Meta Biomed, Japan) (positive control), 2 ppm concentration of nano-curcumin pulpal paste, 4 ppm concentration of nano-curcumin pulpal paste, 6 ppm concentration of nano-curcumin pulpal paste, 8 ppm concentration of nano-curcumin pulpal paste, and ZOE in 1:2 ratio (positive control), and one empty tube as the control group.

### Animal Testing

The rats were anesthetized by injection of 0.4 mL/kg ketamine and 0.02 mL/kg xylazine and their back was shaved and disinfected with betadine. Next, 7 separate incisions were made subcutaneously with adequate distance from each other. Polyethylene tubes were filled with the respective materials (controls tubes remained empty) and implanted subcutaneously according to the order shown in Figure-1, and sutured with absorbable chromic catgut suture thread. During the recovery phase, 10% dextrose was injected to rats intraperitoneally.

The rats were sacrificed after 15, 30, and 60 days [23][26][27] (n=10 rats at each time point) by placing them in a CO2 chamber. The tubes along with 1 cm of the surrounding tissue were resected (Figure-2) and placed in capped plastic containers coded 1 to 210, which contained 10% formalin, for 48 hours. The specimens were then embedded in paraffin. Paraffin blocks were sectioned into 5-µm slices by a microtome, mounted on glass slides, and stained with hematoxylin and eosin. A minimum of 10 slides were prepared from each specimen, and inspected under a microscope (Labomed Leica Galen III, USA) equipped with a digital camera (SSC-DC-58AP, Japan) at x40, x100, x200, and x400 magnifications by an oral and maxillofacial pathologist.

### Histopathological Analysis

Four variables were evaluated in histopathological analysis: (I) presence/absence of calcification, (II) presence/absence of necrosis, (III) thickness of the formed fibrotic capsule, (IV)

degree of inflammation.

Presence/absence of calcification: Presence/absence of calcified tissue was dichotomized and reported as present/absent.

Presence/absence of necrosis: Presence/absence of necrosis was dichotomized and reported as present/absent.

Thickness of the formed fibrotic capsule: The thickness of fibrotic capsule was also scored as follows [28]:

- <150 µm: thin

- >150 µm: thick

Inflammation: The number of inflammatory cells (lymphocytes, plasmacytes, polymorphonuclears, macrophages, and giant cells) was counted in 10 separate fields of each specimen at x400 magnification. The observer was blinded to the type of material and time of sacrifice. The mean number of cells counted in the 10 fields was calculated and scored as follows [28]:

- 0: No inflammatory cell

- 1: <25 inflammatory cells: Mild reaction

- 2: Between 25-125 inflammatory cells: Moderate reaction

- 3: >125 inflammatory cells: Severe reaction

### Statistical Analysis

Data were analyzed by SPSS version 24 (SPSS Inc., IL, USA) using the Chi-square, Mann-Whitney, and Kruskal-Wallis tests (considering the non-normal distribution of data as confirmed by the Kolmogorov-Smirnov test). Level of statistical significance was set at 0.05.

## Results

**Table:** Table1. Presence/Absence of Calcification in the Study Groups at Different Time Points

**Time/Calcification**				**Groups **				
	Metapex	2ppm NPP	4ppm NPP	6 ppm NPP	8 ppm NPP*	ZOE**	control	
	Absent	10	9	10	8	10	9	10
Day 15		%100	%90	%100	%80	%100	%90	%100
	Present	0	1	0	2	0	1	0
		%0.0	%10	%0.0	%20	%0.0	%10	%0.0
	Absent	10	7	8	10	9	9	9
Day 30		%100	%70	%80	%100	%90	%90	%90
	Present	0	3	2	0	1	1	1
		%0.0	%30	%20	%0.0	%10	%10	%10
	Absent	10	9	10	9	10	10	9
Day 60		%100	%90	%100	%90	%100	%1000	%90
	Present	0	1	0	1	0	0	1
%0.0	%10	%0.0	%10	%0.0	%0.0	%10

**NPP:** Nano-curcumin pulpal paste; **ZOE:** Zinc oxide eugenol

**Table:** Table2. Inflammation Score of the Groups at 15 Days (n=10)

**Groups**	**Mean**	**Median**	**Std. error**	**Std. deviation**	**Minimum**	**Maximum**
Metapex	2.1	2	0.233	0.738	1	3
2 ppm nano-curcumin	2.4	2.5	0.221	0.699	1	3
4 ppm nano-curcumin	2.4	2	0.163	0.516	2	3
6 ppm nano-curcumin	2.7	3	0.153	0.483	2	3
8 ppm nano-curcumin	2.6	3	0.221	0.699	1	3
ZOE	1.8	2	0.133	0.422	1	2
Control	1.1	1	0.1	0.316	1	2

**ZOE:** Zinc oxide eugenol

### Presence/Absence of Calcification

Table-1 shows presence/absence of calcification in the study groups at different time points. The Chi-square test showed no significant difference regarding calcification among the groups at 15 (P=0.331), 30 (P=0.342), or 60 (P=0.652) days.

### Presence/Absence of Necrosis

None of the groups showed any sign of tissue necrosis at any time point (P>0.05).

Thickness of fibrotic capsule:

The fibrotic capsule was thin in all groups at all time points (P>0.05).

### Degree of Tissue Inflammation

At 15 days: Table-2 shows the inflammation score of the groups at 15 days. The lowest mean score of inflammation was noted in the control group, and the highest in 6 ppm nano-curcumin group. The difference in inflammation score was significant among the groups (Kruskal-Wallis test, P<0.001). Pairwise comparisons showed that the mean score of inflammation in all experimental groups was significantly higher than that in the control group (P<0.05). Also, the mean inflammation score in 6 ppm nano-curcumin group was significantly higher than that in the ZOE group (P=0.003). No other significant differences were found (P>0.05).

At 30 days: Table-3 shows the inflammation score of the groups at 30 days. The lowest mean score of inflammation was noted in the control group, and the highest in 4 ppm nano-curcumin group. The difference in inflammation score was significant among the groups (Kruskal-Wallis test, P=0.002). Pairwise comparisons showed that the mean score of inflammation in all experimental groups was significantly higher than that in the control group (P<0.05). Also, the mean inflammation score in 4, 6, and 8 ppm nano-curcumin groups was significantly lower than that in the ZOE group.

At 60 days: Table-4 shows the inflammation score of the groups at 60 days. The lowest mean score of inflammation was noted in the control group, and the highest in Metapex group. The difference in inflammation score was significant among the groups (Kruskal-Wallis test, P=0.010). Pairwise comparisons showed that the mean score of inflammation in all experimental groups, except for 2 ppm nano-curcumin (P=0.052) and ZOE (P=0.315), was significantly higher than that in the control group (P<0.05). Also, the mean score of inflammation in Metapex group was significantly higher than that in the ZOE group (P>0.043).

### Trend of Change in Inflammation

Irrespective of the type of material, inflammation gradually decreased from day 15 to day 60 such that at 60 days, no score 3 inflammation was seen in any group.

## Discussion

**Table:** Table3. Inflammation score of the groups at 30 days (n=10)

**Groups**	**Mean**	**Median**	**Std. error**	**Std. deviation**	**Minimum**	**Maximum**
Metapex	1.7	2	0.213	0.675	1	3
2 ppm nano-curcumin	1.8	2	0.249	0.789	1	3
4 ppm nano-curcumin	2.3	2	0.213	0.675	1	3
6 ppm nano-curcumin	2.2	2	0.249	0.789	1	3
8 ppm nano-curcumin	2.1	2	0.233	0.738	1	3
ZOE	1.3	1	0.26	0.823	0	3
Control	0.9	1	0.233	0.738	0	2

**ZOE:** Zinc oxide eugenol

**Table:** Table4. Inflammation Score of the Groups at 60 Days (n=10)

**Groups**	**Mean**	**Median**	**Std. error**	**Std. deviation**	**Minimum**	**Maximum**
Metapex	1.6	2	0.163	0.516	1	2
2 ppm nano-curcumin	1.3	1	0.153	0.483	1	2
4 ppm nano-curcumin	1.4	1	0.163	0.516	1	2
6 ppm nano-curcumin	1.4	1	0.163	0.516	1	2
8 ppm nano-curcumin	1.4	1	0.163	0.516	1	2
ZOE	1	1	0.149	0.471	0	2
Control	0.7	1	0.153	0.483	0	1

This study assessed the biocompatibility of different concentrations of a nano-curcumin pulpal paste, in comparison with ZOE and Metapex in rats. The results showed no necrosis at any time point. The observed calcifications were not significant either. The thickness of the formed fibrotic capsule was thin in all groups at all time points. The degree of inflammation gradually decreased in all groups from day 15 to day 60 such that at day 60, no grade III inflammation was seen in any group. At 15 days, all experimental groups showed significantly higher degree of inflammation than the control group. However, among the experimental groups, only the difference between 6 ppm nano-curcumin and ZOE was significant (lower inflammation in the ZOE group).

At 30 days, all experimental groups showed significantly higher degree of inflammation than the control group, except for ZOE. Also, significant differences were found between nano-curcumin groups with the ZOE group. At 60 days, the control group showed significantly lower inflammation than all other groups except for ZOE. Among the experimental groups, only Metapex had a significant difference with ZOE. Although the Metapex group showed generally higher inflammation than the ZOE group, this difference was only significant at 60 days.

Al-Ostwani et al, [29] in their clinical study showed comparable success rate of pulpectomy with ZOE and Metapex in primary teeth, and added that their difference was in their resoprtion rate. ZOE is resorbed slower than the rate of physiological root resorption while Metapex is resorbed faster. Gupta and Das [30] reported the success rate of ZOE and Metapex in pulpectomy of primary teeth to be 85.7% and 90.4%, respectively, and added that preoperative symptoms (pain, swelling, and sensitivity to percussion) resolved faster in the Metapex group. Reddy and Ramakrishna [31] showed that ZOE had significantly higher antimicrobial activity than Metapex, such that no bacterial growth inhibition zone was noted around Metapex. In the present study, inflammation decreased with time, such that score 3 was not seen in any group at 60 days. This finding was in line with the results of Onay et al, [32] who reported a reduction in inflammation 1 week after implantation of different materials in the connective tissue of rats. Some other studies reported similar results [27][33].

The degree of inflammation of the control group was significantly lower than other groups. Low score of inflammation in this group observed at 15 days can be due to surgical trauma, and inflammation at 30 and 60 days can be due to mechanical irritation of tissue by the borders of polyethylene tubes [27]. At 15 days, moderate to severe inflammation was noted in all groups except for the control group in the present study. Pilownic et al. [22] compared the biocompatibility of ZOE, Vitapex, Calen paste, and MTA-based materials in rats. They reported moderate to severe inflammation in the first 15 days, and moderate inflammation at 30 days. Mild to moderate inflammation was noted in ZOE-based groups at 60 days. Their results in this regard where in agreement with the present findings. However, they found calcifications around ZOE at 60 days, which was in contrast to the present findings.

Curcumin has anti-inflammatory, antibacterial, antiviral, antifungal, anti-diabetes, and anti-coagulant properties [34]. It exerts its anti-inflammatory effects by inhibiting the production of tumor necrosis factor alpha, and interleukin 1, which are released from monocytes and macrophages, and play an important role in modulation of inflammatory processes. Also, curcumin increases the migration of fibroblasts, granulation tissue formation, and collagen deposition. Moreover, curcumin increases the production of transforming growth factor-beta and enhances the proliferation of fibroblasts and accelerates wound healing [34]. Hugar et al. [35] evaluated the clinical and radiographic success rate of pulpotomy with curcumin, in comparison with formocresol, CH, and propolis in primary molars and showed their comparable efficacy. Also, Purhit et al. [36] used turmeric powder for pulpotomy of primary teeth and reported a success rate of 93.4% after 6 months. Prabhakar et al. [37] used curcumin in comparison with MTA for pulpotomy of rats. They evaluated dentinal bridge formation, number of inflammatory cells, and soft tissue organization after 7, 14, and 30 days. They also reported a reduction in inflammatory cells from day 7 to day 30, which was in agreement with the present findings. They observed the formation of dentinal bridge in the MTA group and reported that its thickness gradually increased up to 30 days. However, the formed dentinal bridge was insignificant in the curcumin group and its thickness did not change within 30 days. This finding was also in agreement with the present results despite the fact that they evaluated pulpal cells while subcutaneous connective tissue was evaluated in the present study. As mentioned earlier, nano-curcumin has significant advantages compared with curcumin. It has better water solubility, and subsequently higher bioavailability and biological activity [14]. Also, it is more stable and less sensitive to light and oxygen [38].

It has better absorption, and can accelerate wound healing [39]. Thus, nano-curcumin paste was selected for evaluation in the present study. Also, Barja-fidalgo et al, [5] in their systematic review showed that ZOE and iodoform plus CH paste are suitable materials for filling of primary root canals. Thus, they were selected as the positive control groups in the present study for the purpose of comparison with different concentrations of nano-curcumin paste. As mentioned earlier, all concentrations of nano-curcumin showed significantly higher inflammation than ZOE at 30 days, except for 2 ppm concentration, which showed no significant difference with ZOE at any time point. Thus, it may be stated that 2 ppm concentration was the best concentration of nano-curcumin for further experimentation, since it yielded results comparable to ZOE and Metapex in terms of tissue reaction and biocompatibility.

In the present study, inflammation was classified based on the number of inflammatory cells [33]. However, this quantitative classification can show a significant difference only when the difference among the groups is very large [33]. This statement may explain lack of a significant difference among the experimental groups in the present study. On the other hand, it should be noted that qualitative assessment of tissue inflammation is not a precise method for the comparison of degree of inflammation of different materials or one single material at different time points.

The thickness of fibrotic capsule around the tubes was also evaluated in the present study, which was thin in all groups at all time points. Pilownic et al. [22] noticed a thick fibrotic capsule around the materials at 15 days, which became thin at 60 days. Sanders and Rochefort [40] showed a significant correlation between the capsule thickness and degree of inflammation. Thinner fibrotic capsules indicated higher biocompatibility of the tested materials. Queiroz et al. [24] demonstrated a reduction in thickness of granulomatous tissue around ZOE, Calen/ZO paste, and Sealapex in rats after 7 to 63 days.

The present study was an animal study to assess tissue reaction and biocompatibility of nano-curcumin pulpal paste. Further in vitro and clinical studies are required on its rate of resorption in primary root canals, tooth discoloration potential, effect on microhardness, and clinical and radiographic success when used as root filling material in primary teeth.

## Conclusion

All tested concentrations of nano-curcumin pulpal paste were biocompatible, compared with the positive controls; but 2 ppm concentration was the most biocompatible. Within the limitations of this in vitro study, 2 ppm concentration of nano-curcumin may be suggested for further experimentation and possible clinical use.

## Conflict of Interests

None declared.
